# Exploring the potential of *Scabiosa columbaria* in Alzheimer's disease treatment: An *in silico* approach

**DOI:** 10.1016/j.jtumed.2024.09.003

**Published:** 2024-09-19

**Authors:** Riyan A.P. Irsal, Gusnia M. Gholam, Maheswari A. Dwicesaria, Tiyara F. Mansyah, Fernanda Chairunisa

**Affiliations:** a Biomatics, Bogor, West Java, Indonesia; b Department of Biochemistry, Faculty of Mathematics and Natural Sciences, Bogor Agricultural University, Bogor, Indonesia; c Bioinformatics Research Center, Indonesian Institute of Bioinformatics, Malang, Indonesia; d Universitas Nasional, Department of Biology, South Jakarta, Indonesia

**Keywords:** إنزيم أسيتيل كولين إستيراز, إنزيم شطر البروتين السلائف للأميلويد بيتا 1, عامل طبيعي مضاد لمرض الزهايمر, إنزيم محول عامل نخر الورم ألفا, برنامج ياسارا ستركتشر, Acetylcholinesterase, BACE1, Natural anti-AD agent, TACE, YASARA structure

## Abstract

**Objectives:**

Alzheimer's disease (AD) is posing an increasing global threat and currently lacks effective treatments. Therefore, this study was aimed at exploring phytochemicals in *Scabiosa columbaria* (*S. columbaria*) as inhibitors of acetylcholinesterase (AChE), β-site APP cleavage enzyme 1 (BACE1), and TNF-α converting enzyme (TACE) in AD. *S. columbaria* contains various bioactive compounds, such as chlorogenic acid, linalool, and catechins, which are known for their detoxification properties, capacity to resist and manage harmful moisture buildup, and therapeutic roles in COVID-19. Several studies have also shown that *S. columbaria* extract has strong antioxidant activity, and may potentially decrease neuroinflammation in AD. Therefore, this study investigated the interactions between *S. columbaria* phytochemicals and key enzymes associated with AD, thus providing opportunities for the development of new therapeutic candidates.

**Methods:**

A total of 27 phytochemicals were evaluated for their inhibitory activity against AChE, BACE1, and TACE with YASARA Structure. ADMET profiles and toxicity were assessed. The top candidate compounds underwent 100 ns MD simulations.

**Results:**

All ligands met Lipinski's rule and showed low toxicity. Catechins, compared with the known drug galantamine, showed higher inhibitory activity and interacted with additional active sites on AChE, thus suggesting potentially higher efficacy. Moreover, chlorogenic acid showed stronger inhibitory activity against TACE than the control drug (aryl-sulfonamide), thereby suggesting a different mechanism of action. MD simulation revealed that the formed complexes had good stability. However, further exploration is necessary.

**Conclusion:**

*S. columbaria* derivative compounds are promising drug candidates because of their properties, including the affinity of chlorogenic acid toward TACE and hydrogen bond enhancing ligand–receptor interactions. MD simulation indicated stable ligand–protein complexes, and the radius of gyration and MM-PBSA calculations revealed favorable binding and interaction energies. Our findings demonstrate the identified compounds' potential for further drug development.

## الملخص

**أهداف البحث:**

يعد مرض الزهايمر تهديدا عالميا متزايدا، ولا يوجد علاج فعال له حاليا. ولذلك، تهدف هذه الدراسة إلى استكشاف المركبات النباتية في نبات الجرباء العمودية كمثبطات لإنزيم أسيتيل كولين إستيراز، وإنزيم شطر البروتين السلائف للأميلويد بيتا 1، وإنزيم محول عامل نخر الورم ألفا في مرض الزهايمر. يحتوي نبات الجرباء العمودية عادة على مركبات نشطة بيولوجيا مختلفة، مثل حمض الكلوروجينيك، واللينالول، والكاتيكين، المعروفة بخصائصها في إزالة السموم، والقدرة على التغلب على احتباس الرطوبة السامة، والآليات العلاجية في كوفيد-19. وقد أظهرت العديد من الدراسات أيضا أن المستخلص له نشاط مضاد للأكسدة قوي، مما قد يقلل من الالتهاب العصبي في مرض الزهايمر. في هذا السياق، توفر هذه الدراسة الحالية فرصا لتطوير المواد الكيميائية النباتية لنبات الجرباء العمودية كمرشحين علاجيين جدد لمرض الزهايمر من خلال دراسة التفاعلات مع الإنزيمات الرئيسية.

**طرق البحث:**

تم تقييم 27 مركبا نباتيا للنشاط التثبيطي ضد الإنزيمات المستهدفة باستخدام برنامج "ياسارا ستركتشر". تم تقييم الخصائص الدوائية والحركية والأيضية والسمية للمركبات. خضعت المركبات الأعلى تصنيفا لمحاكاة ديناميكية جزيئية لمدة 100 نانوثانية.

**النتائج:**

استوفت جميع المركبات معايير "ليبينسكي" مع انخفاض كبير في السمية. أظهرت الكاتيكينات نشاطا تثبيطيا أكبر وتفاعلات إضافية مع إنزيم أسيتيل كولين إستيراز مقارنة بدواء الجالانتامين. أظهر حمض الكلوروجينيك نشاطا تثبيطيا أقوى ضد إنزيم محول عامل نخر الورم ألفا مقارنة بدواء المراقبة. أظهرت المحاكاة الديناميكية الجزيئية استقرارا جيدا للمعقدات المتكونة، ولكنها تتطلب المزيد من الاستكشاف.

**الاستنتاجات:**

أظهرت المركبات المشتقة من نبات الجرباء العمودية إمكانات واعدة كمرشحات دوائية لمرض الزهايمر، خاصة حمض الكلوروجينيك في تفاعله مع إنزيم محول عامل نخر الورم ألفا. أكدت المحاكاة الديناميكية الجزيئية وحسابات الطاقة استقرار وقوة الروابط بين المركبات والبروتينات المستهدفة، مما يشير إلى إمكانية تطوير هذه المركبات كعلاجات محتملة لمرض الزهايمر.

## Introduction

Alzheimer's disease (AD) is a prevalent neurodegenerative disease posing substantial global health challenges involving cognitive decline and memory impairment. AD accounts for 60%–80% of all neurodegenerative cases.^1^ In the context of the aging population and increasing AD prevalence, developing new therapeutic methods is important.^1^^,^^2^ Current AD treatments are primarily symptomatic, and only four FDA-approved drugs—donepezil, memantine, galantamine, and rivastigmine—are available.^3^ Despite their potential, these single-compound drugs have limited effectiveness and many adverse effects.^4^ Given the limitations of the current therapeutic options, an urgent need exists for new disease-modifying treatments.^5^ An effective option in this context is traditional Chinese medicine (TCM), which uses a variety of natural products with potential benefits for various conditions.

This study therefore was aimed at demonstrating the potential of *Scabiosa columbaria* (*S. columbaria*), a natural product, as a promising anti-AD agent. *S. columbaria* contains a diverse range of bioactive compounds, including chlorogenic acid, linalool, and catechin, which are known in TCM for their potential health benefits.^6^, ^7^, ^8^ Linalool is often used in prescriptions to accumulation of harmful moisture and toxins in patients with pulmonary syndrome, whereas catechins are used as a therapeutic in severe cases of COVID-19.^8^ Chlorogenic acid has substantial potential as an alternative treatment for a variety of neurological diseases, including AD, neuropathic pain, post-traumatic stress disorder, and Parkinson's disease. In herbal preparations for heat relief and detoxification in TCM, chlorogenic acid from plants significantly affects IL-4 and IL-13 signaling pathways, ESR-mediated signaling, and extranuclear estrogen signaling.^9^, ^10^, ^11^, ^12^ Moreover, this compound can cross the blood–brain barrier and treat specific neurological disorders.^13^ Chlorogenic acid treatment after transient total cerebral ischemia also ameliorates memory loss and decreases hippocampal cell death.^14^ Recent studies have indicated that linalool produces antidepressant-like effects through interaction with the serotonergic pathway. *In vivo* AD experiments have indicated that this compound decreases neurotoxicity and improves cognitive function by activating Nrf2/HO-1 and BDNF.^15^, ^16^, ^17^ Catechin also has diverse molecular mechanisms in AD pathways, and have shown been shown to have a high ability to cross the blood–brain barrier, increase antioxidation, and inhibit lipid peroxidation by increasing Nrf2 protein expression.^18^

According to previous studies, *S. columbaria* extracts show substantial antioxidant FRAP activity, thereby suggesting potential neuroprotective effects against the inflammatory mechanisms in AD. The methanol extract has FRAP activity of 200 μg/mL, nearly equivalent to that of 13 μg/mL Trolox. The flowers and leaves of *S. columbaria* also have high antioxidant activity (DPPH), with IC_50_ values of 0.0114 and 0.0138 (μg/mL).^6^^,^^19^ Therefore, several compounds in *S. columbaria* may serve as promising treatments for neurodegenerative disease. Recent progress in drug development for AD and understanding of the molecular mechanisms underlying the disease has led to the identification of key enzymes as potential targets for intervention. These targets include acetylcholinesterase (AChE), β-site APP cleaving enzyme 1 (BACE1), and TNF-α converting enzyme (TACE), each of which has an essential role in AD pathology progression. Inhibiting BACE1, might potentially slow the formation and progression of amyloid plaques, whereas TACE inhibition might decrease neuroinflammation, another key pathological feature of AD. Several studies have shown that inhibiting AChE enhances cholinergic neurotransmission, which is impaired in the disease. These enzymes were selected in this current study, on the basis of current understanding of AD pathogenesis. Although many other targets are associated with AD, the three selected targets are well established and have been widely explored, thus providing a strong foundation for further study and development.^19^^,^^20^

**Figure 1:**
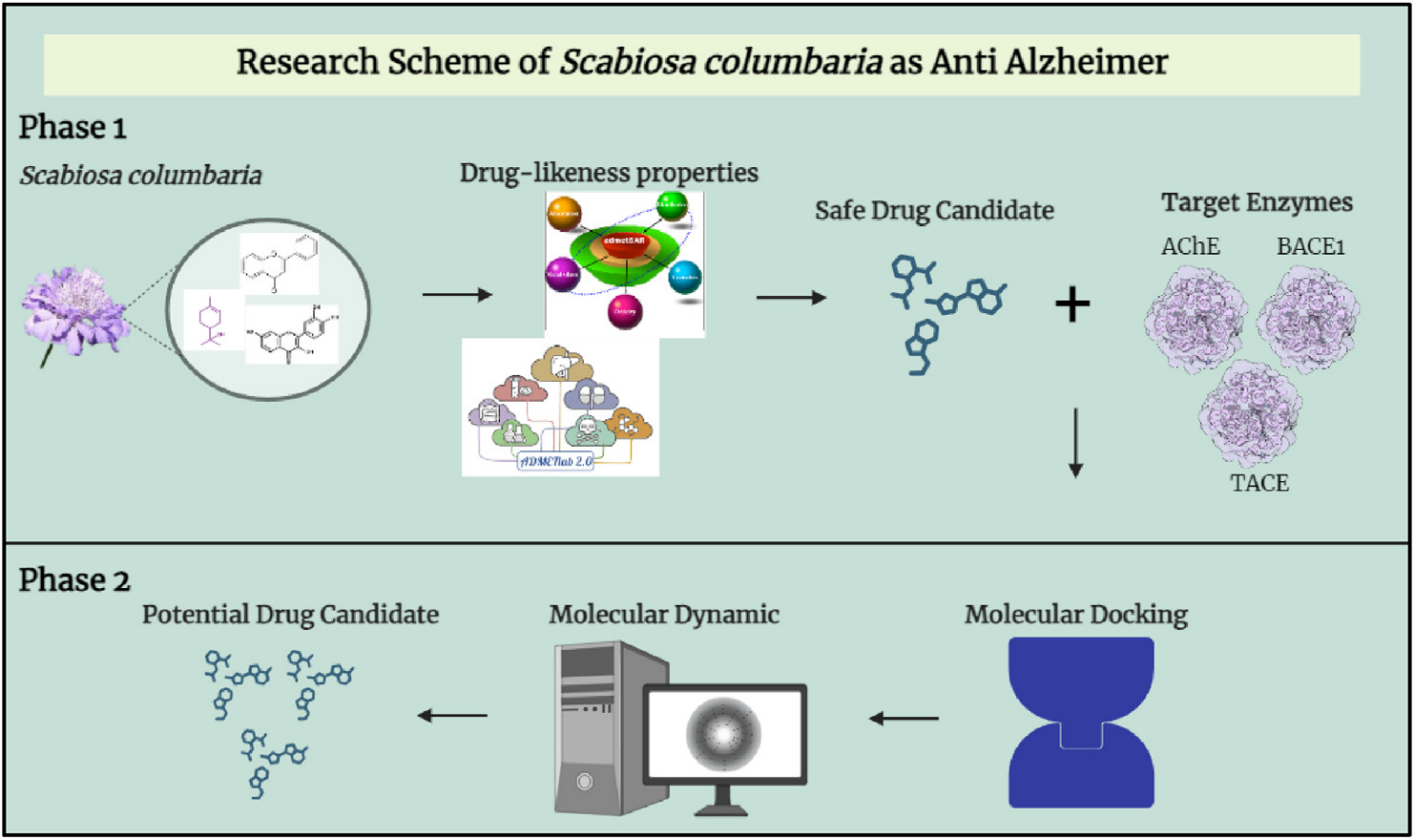
Research scheme for predicting *Scabiosa columbaria* compounds as anti-Alzheimer's disease agents.

To examine the roles of the key enzymes AChE, BACE1, and TACE in the development of AD, this study focused on *in silico* analysis of *S. columbaria* compounds' binding affinity toward, and interactions with, these enzymes.^20^^,^^21^ Inhibition of AChE has been suggested to improve cholinergic neurotransmission and potentially decrease cognitive decline.^21^ Moreover, BACE1 is essential for the formation of amyloid beta peptide (Aβ), a component of the neurotoxic plaques that are hallmarks of AD. Targeting BACE1 offers a strategy to prevent or slow the accumulation of Aβ peptides. TACE, which is involved in neuroinflammation, is associated with the release of pro-inflammatory cytokines, another prominent feature of AD pathology.^20^ To investigate these enzymes as targets, we used an *in silico* approach to explore the potential of *S. columbaria* as a natural therapeutic candidate. The results are expected to provide opportunities for the development of novel therapeutic strategies using natural compounds of *S. columbaria* to target specific enzymatic pathways in AD, thereby potentially improving clinical outcomes. The scheme of this study is shown in Figure 1.

## Materials and Methods

### Preparation of ligands and receptors for *in silico* study of *S. columbaria*

This study used the chemical composition of *S. columbaria* based on Akar^6^ as a potential herbal medicine.^6^ The three-dimensional structures of the herbal medicine and SMILES were obtained from PubChem (https://pubchem.ncbi.nlm.nih.gov/) (Table 1). In addition, YASARA Structure was used to optimize all ligands and obtain the molecular structures with the lowest free energy. Water and nonessential residues were then removed for enzyme preparation, and hydrogen atoms were added. Subsequently, bond orders and hydrogens were adjusted according to a pH of 7.4. The structures of AChE in PDB ID: 4EY6,^22^ BACE1 in PDB ID: 4DJU,^23^ and TACE in PDB ID: 2OI0^24^ were extracted from the Protein Data Bank (PDB; https://www.rcsb.org/).^25^

**Table 1:** List of Ligands from *Scabiosa columbaria*.

Ligands	SMILES	CID	Source
Gallic acid	C1=C(C=C(C(=C1O)O)O)C(=O)O	370	Akar^6^
Catechin	C1C(C(OC2=CC(=CC(=C21)O)O)C3=CC(=C(C=C3)O)O)O	9064	
Chlorogenic acid	C1C(C(C(CC1(C(=O)O)O)OC(=O)C=CC2=CC(=C(C=C2)O)O)O)O	1794427	
4-OH benzoic acid	CN(C)C1=CC=C(C=C1)N=NC2=CC=C(C=C2)O	97486	
Caffeic acid	C1=CC(=C(C=C1C=CC(=O)O)O)O	689043	
Linalool	CC(=CCCC(C)(C=C)O)C	6549	
p-Anisyl isonitrile	COC1=CC=C(C=C1)[N+]#C	6329374	
o-Toluidine, 5-isopropyl-	CC1=C(C=C(C=C1)C(C)C)N	137414	
1-Tetradecanol	CCCCCCCCCCCCCCO	8209	
4-Hexylacetophenone	CCCCCCC1=CC=C(C=C1)C(=O)C	123462	
5-Octadecenal	CCCCCCCCCCCCC=CCCCC=O	545652	
4-Octadecenal	CCCCCCCCCCCCCC=CCCC=O	5365018	
2-Octadecoxyethanol	CCCCCCCCCCCCCCCCCCOCCO	75050	
Nonacosane	CCCCCCCCCCCCCCCCCCCCCCCCCCCCC	12409	
Verbenene	CC1(C2CC1C(=C)C=C2)C	6427476	
a-Terpineol	CC1=CCC(CC1)C(C)(C)O	443162	
Carvone	CC1=CCC(CC1=O)C(=C)C	7439	
Thymol	CC1=CC(=C(C=C1)C(C)C)O	6989	
p-Eugenol	COC1=C(C=CC(=C1)CC=C)O	3314	
b-Ionone	C1=C(C(CCC1)(C)C)C=CC(=O)C	638014	
Caryophyllene oxide	CC1(CC2C1CCC3(C(O3)CCC2=C)C)C	1742210	
Palustrol	CC1CCC2(C1C3C(C3(C)C)CCC2C)O	110745	
8-Cedren-13-ol	CC1CCC2C13CC=C(C(C3)C2(C)CO)C	519545	
Benzophenone	C1=CC=C(C=C1)C(=O)C2=CC=CC=C2	3102	
a-Curcumene	CC1=CC=C(C=C1)C(C)CCC=C(C)C	92139	
6,10,14-Trimethyl-2-pentadecanone	CC(C)CCCC(C)CCCC(C)CCCC(=O)C	10408	

### Analysis of drug-likeness properties

Lipinski's rule of five is an important drug-likeness test used to evaluate the potential of a chemical compound to be an orally active drug. This method indicated that all ligands from *S. columbaria* were safe and could potentially be used as drug molecules. ADMETLab2.0 (https://www.swissadme.ch/) and Admetsar1 (http://lmmd.ecust.edu.cn/admetsar1/home/) were used to analyze drug-likeness properties.^26^^,^^27^

### Molecular docking study of *S. columbaria* against AD

Molecular docking analyses were conducted in YASARA Structure (Bioinformatics 30, 2981-2982 version 19.9.17). The YASARA Structure docking procedure was initiated through the script “dock_runscreening.mcr,” by using the VINA docking engine. Each docking simulation was repeated 100 times with the AMBER 14 forcefield, and the simulation cell was filled with water set to a density of 0.997 g/L. Subsequently, Gasteiger charges were assigned to model the electrostatic interactions of the molecules. AChE (PDB ID: 4EY6) was docked with a grid size of 19.80 nm × 19.80 nm × 19.80 nm (5 Å cuboid). For BACE1 (PDB ID: 4DJU), a grid size of 16.60 nm × 16.60 nm × 16.60 nm (4.0 Å cuboid) was used. Finally, docking of TACE (PDB ID: 2OI0) was performed with a grid size of 17.80 nm × 17.80 nm × 17.80 nm (4.0 Å cuboid). The top ligand structures for each enzyme were then analyzed in Discovery Studio 2017 R2 Client.^28^

### Molecular dynamics simulation

**Figure 2:**
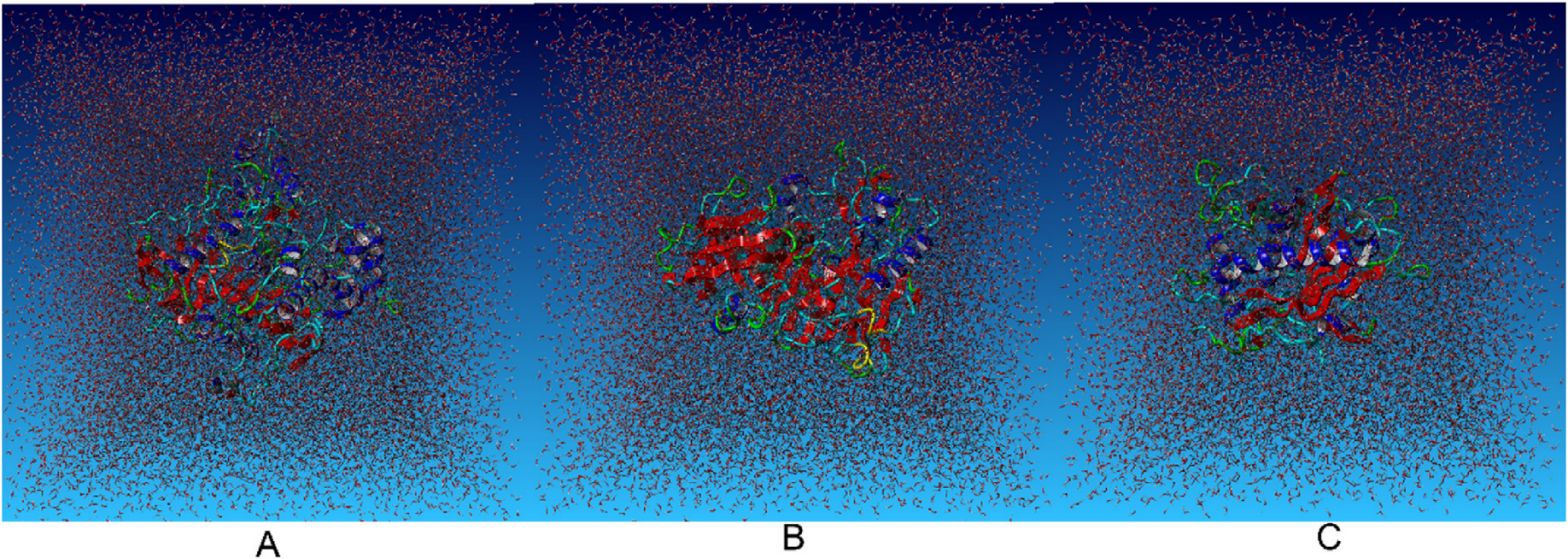
Visualization of the state of the molecular dynamics simulation of A) Catechin-AChE. B) Chlorogenic acid-BACE1. C) Chlorogenic acid-TACE.

Molecular dynamics (MD) simulation was used to investigate the protein–ligand complexes' dynamic behavior and stability for the top-scoring ligands identified for each of the three target enzymes (AChE, BACE1, and TACE). This powerful drug discovery method considers the inherent flexibility of proteins, thus providing a more realistic representation of binding than static docking methods. YASARA Structure (Bioinformatics 30, 2981-2982 version 19.9.17) was used for simulation with the AMBER14 force field. The simulation was conducted under physiological conditions with a constant temperature of 310 K, pH 7.4, and 0.9% NaCl solution. Gasteiger charges were then assigned to model the electrostatic interactions of the molecules. In addition, the simulation cell was solvated with water set to a 0.997 g/L density and extended by 20 Å in each direction to account for solvation effects (Figure 2). The trajectories were generated with the “md_run.mcr” macro, and the entire simulation was run for 100 ns. The complete simulation protocol, including initial energy minimization procedures, was managed through YASARA Structure macros.^29^

### Molecular dynamics stability analysis

The generated simulation trajectories were analyzed to assess the stability of the protein–ligand complexes. Root mean square deviation (RMSD), root mean square fluctuation (RMSF), solvent-accessible surface area (SASA), and radius of gyration (Rg) were calculated with the md_analyze.mcr macro.^30^ Subsequently, ligand binding free energies were evaluated with the md_analyzebindenergy.mcr macro with the molecular mechanics Poisson–Boltzmann surface area (MM-PBSA) method. These macros read the saved previously generated snapshots. Quantitative analysis of the simulation results was performed in Microsoft Excel for data cleaning and graphical visualization.^31^

## Results

### Drug-likeness properties

**Figure 3:**
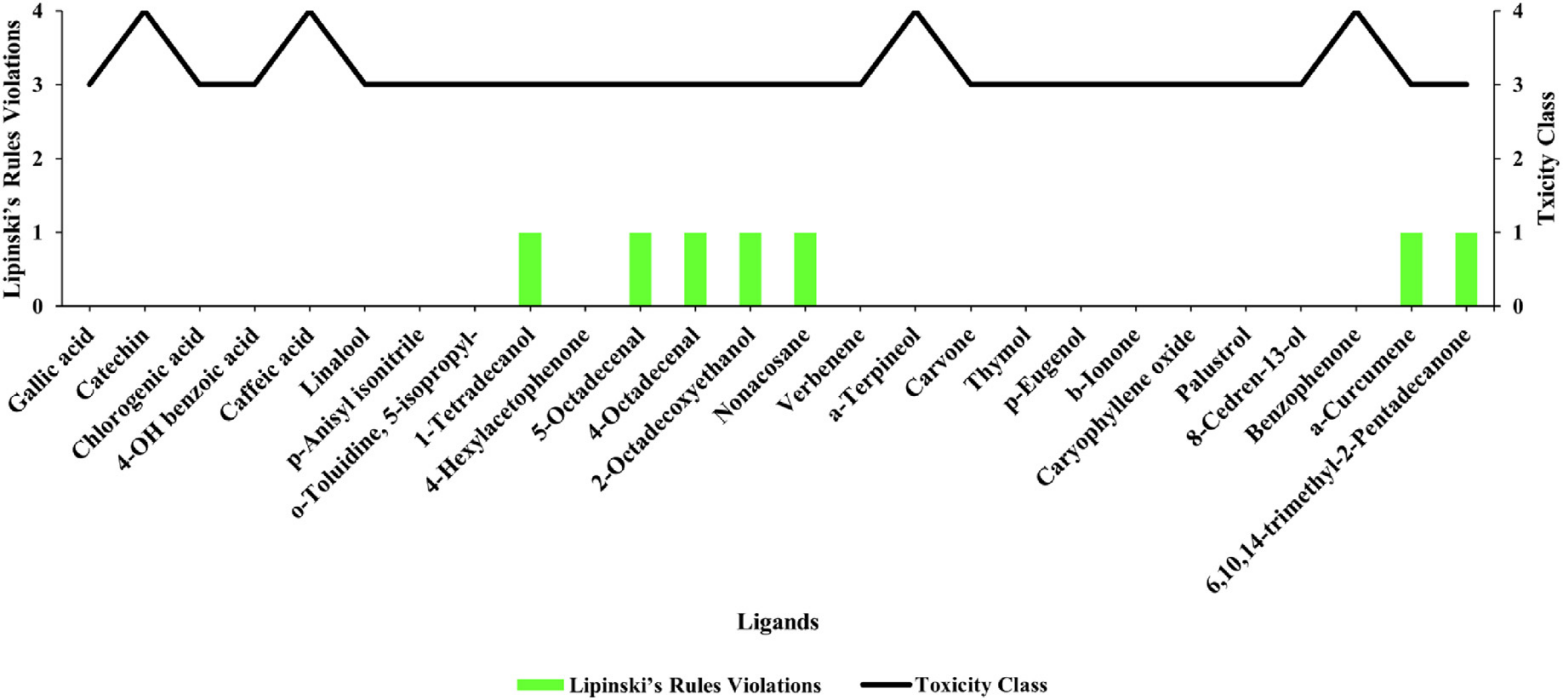
Comparison of Lipinski's rule violation and toxicity class for each ligand.

First, drug-likeness and safety profiles were assessed for 27 candidate phytochemicals. Some phytochemicals violated Lipinski's rule of five, namely logP >3. All chemical compounds of *S. columbaria* satisfying Lipinski's rule of five and having safety classes 3 and 4 were selected for evaluation through molecular docking and dynamic testing (Figure 3). The preliminary stages of drug screening assessed ligand bioavailability and used Lipinski's rule of five as a predictive tool to evaluate the likelihood of a compound's success or failure in metabolism according to its resemblance to known drugs.

### Molecular docking

Molecular docking was performed to demonstrate the detailed interactions between *S. columbaria* compounds and AChE, BACE1, and TACE. The results were visualized with YASARA Structure. Molecular docking of 27 chemical compoounds from *S. columbaria* was also performed against the three enzymes, to identify potential drugs. The binding affinities (kcal/mol) were recorded for the enzymes with the top ten scores. The control drugs had the highest binding affinity to AChE and BACE1, at −10.526 kcal/mol and −8.983 kcal/mol, respectively.

**Figure 4:**
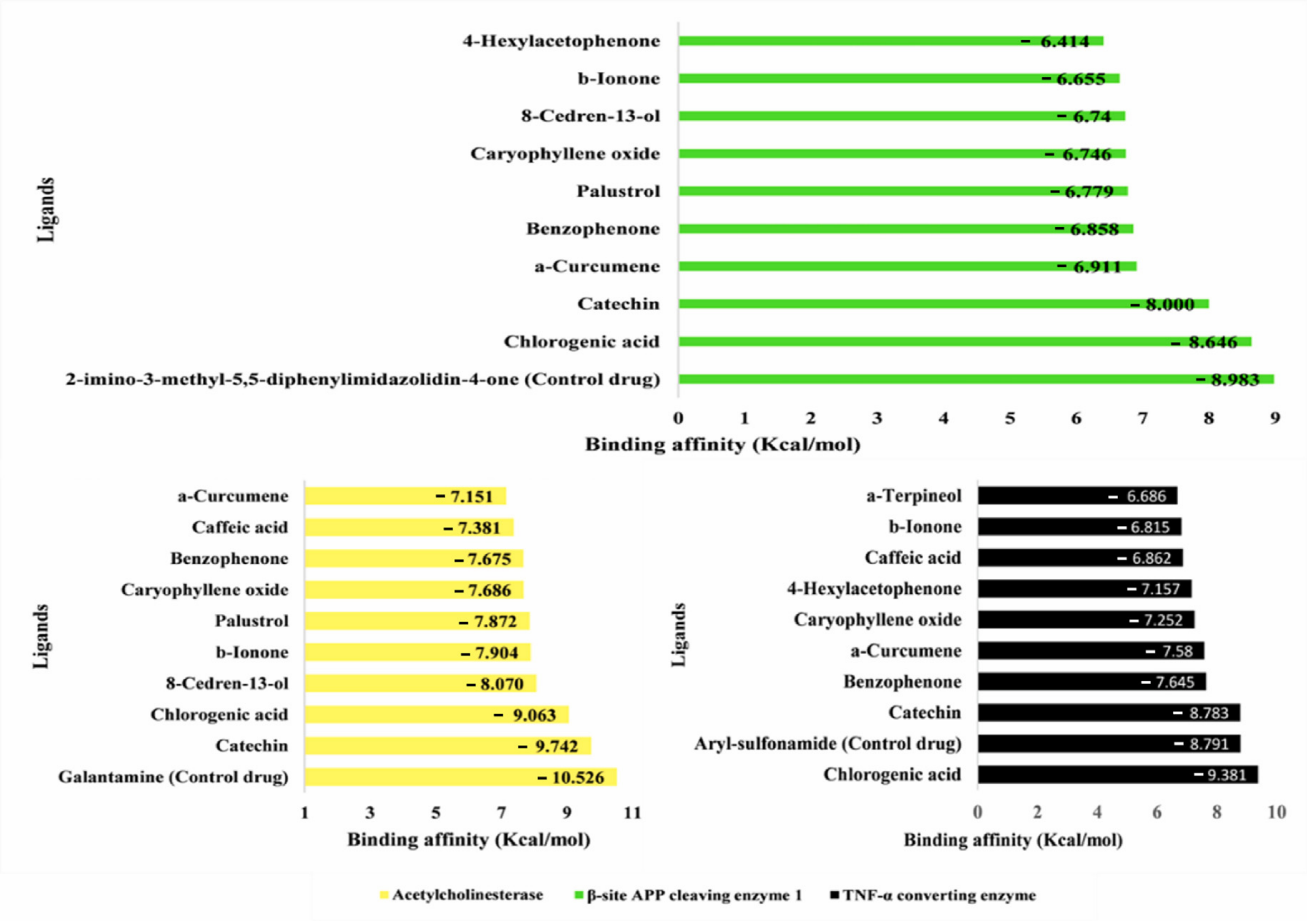
Molecular docking scores of *Scabiosa columbaria* compounds toward three human target enzymes associated with Alzheimer's disease.

Catechin exhibited a binding affinity toward AChE of −9.742 kcal/mol, approaching that of the control drug galantamine (−10.526 kcal/mol). In addition, chlorogenic acid showed the strongest binding affinity toward BACE1 (−9.381 kcal/mol), but this value was not significantly lower than that of the control drug 2-imino-3-methyl-5,5-diphenylimidazolidin-4-one (−8.983 kcal/mol). Chlorogenic acid also displayed a binding affinity toward TACE (−8.646 kcal/mol) near that of the control drug aryl-sulfonamide (−8.791 kcal/mol) (Figure 4).

### Analysis of interactions with multiple enzymes for anti-AD treatment

The interactions of *S. columbaria* compounds with relevant AD targets were visualized and analyzed in Discovery Studio Visualizer. Galantamine, 2-imino-3-methyl-5,5-diphenylimidazolidin-4-one, and aryl-sulfonamide were selected as controls, because of their known binding mechanisms to target structures from the PDB. An in-depth analysis of the binding interactions between the top three compounds and the enzymes AChE, BACE1, and TACE was conducted (Figure 4). Each enzyme had the following distinct active sites: AChE: SER203, GLU334, and HIS447; BACE1: ASP93 and ASP289; and TACE: GLU406.

Figure 6A shows catechin's and galantamine's binding strength and site coverage in interacting with AChE. Catechin binds AChE at two active sites, SER203 and HIS447, thus forming van der Waals interactions. This compound also forms seven interactions with AChE, including five hydrogen bonds (three conventional and two carbon-hydrogen bonds) and two van der Waals interactions. The extensive network of interactions across two active sites suggested a highly stable and specific binding mode. Meanwhile, galantamine showed weaker binding, establishing two active sites (Figure 6B) with one interaction including carbon-hydrogen bonds at HIS447. Galantamine forms two hydrogen bonds, a number fewer than those formed by catechin. In addition, catechin and galantamine form hydrophobic and van der Waals interactions, although these associations do not contribute directly to the specificity of ligand–protein interactions. Chlorogenic acid interacts with TACE via GLU406. The specific binding is mediated by a van der Waals interaction (Figure 6C). Compared with the TACE control drug (aryl-sulfonamide), which forms conventional hydrogen bonds at the same site, chlorogenic acid uses a different binding mechanism (Figure 6D). This result was unexpected, and no apparent interaction with any BACE1 active sites was observed. Further investigation was performed to investigate the nature and potential implications of this binding. The BACE1 control drug 2-imino-3-methyl-5,5-diphenylimidazolidin-4-one showed diverse interactions. Thus, our findings established two conventional hydrogen bonds and interactions with BACE1 active sites (ASP93 and ASP289), thus indicating a broader and potentially more potent binding profile than that of chlorogenic acid (Figure 6F).

**Figure 5:**
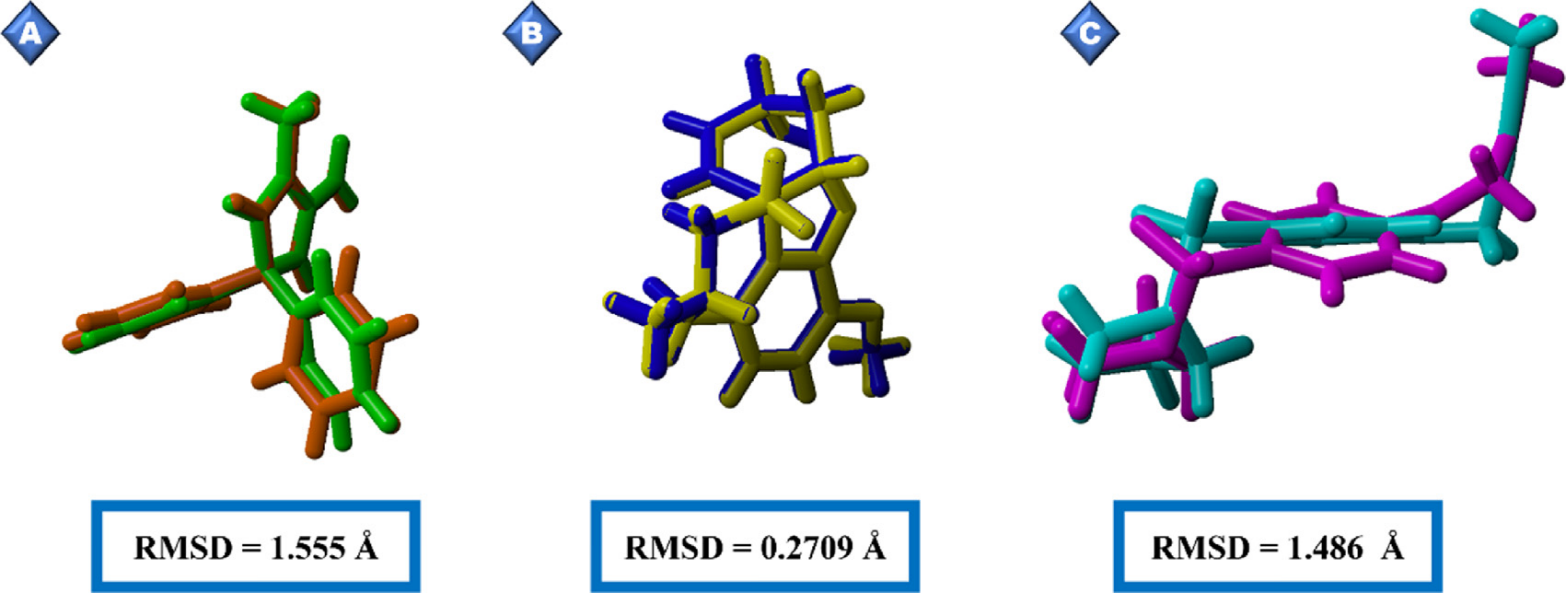
Validation of the grid box for the molecular docking protocol: A) BACE1, re-docked ligand (orange) and native ligand (green); B) AChE, re-docked ligand (yellow) and native ligand (blue); C) re-docked ligand (cyan) and native ligand (purple).

**Figure 6:**
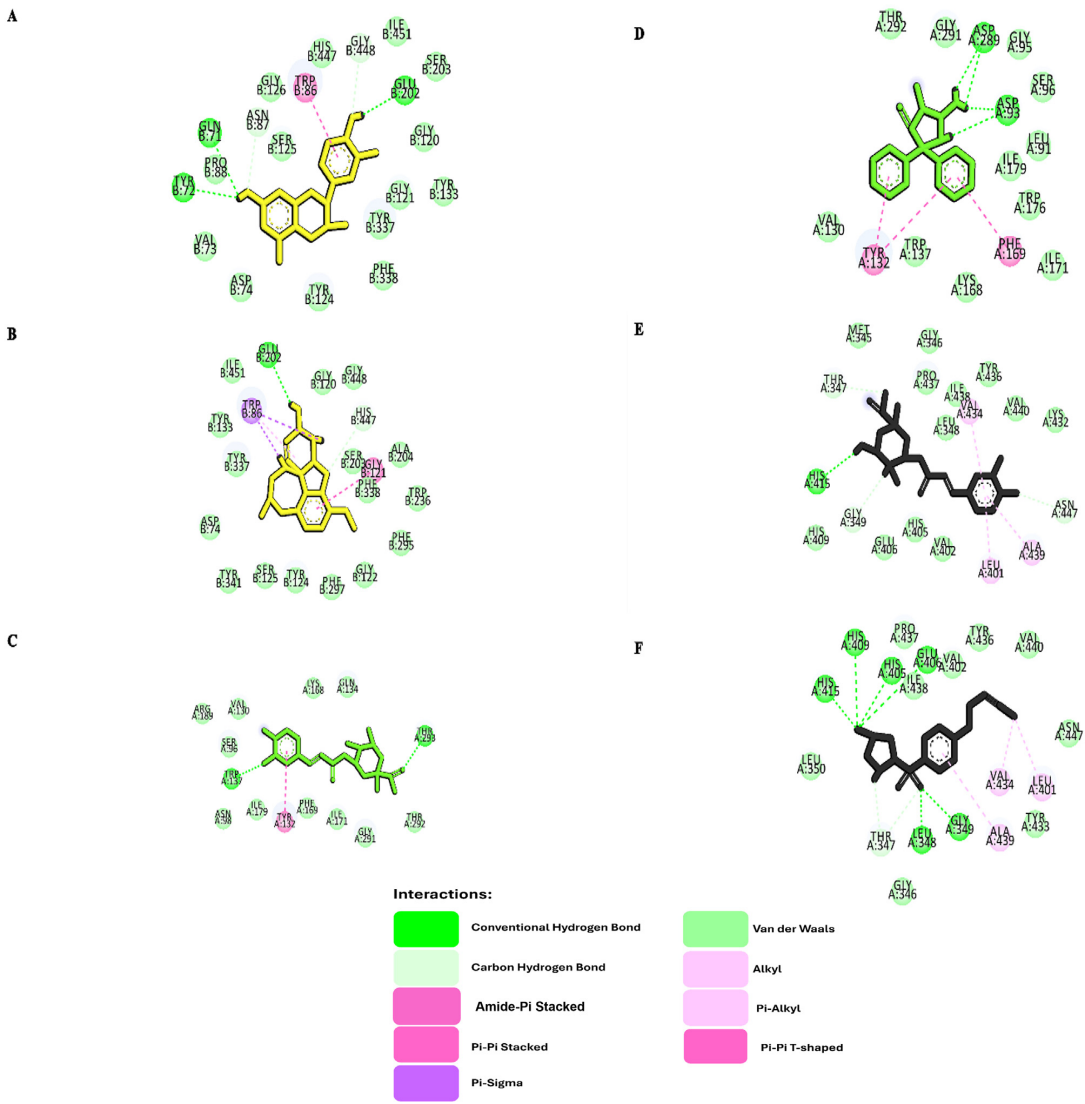
Molecular interactions between ligands and corresponding control drugs targeting key enzyme-related targets in Alzheimer's disease: A) catechin-AChE; B) galantamine (control drug)-AchE; C) chlorogenic acid-BACE1; D) 2-imino-3-methyl-5,5-diphenylimidazolidin-4-one (control drug)-BACE1; E) chlorogenic acid-TACE; F) aryl-sulfonamide (control drug)-TACE.

### Molecular dynamics simulation

MD simulation was performed to compute the stability of ligand–enzyme complexes. Several parameters were used, including RMSD, RMSF, Rg, SASA, and binding free energy calculation with MM-PBSA. The dynamic behavior and stability of six complexes were determined according to RMSD, RMSF, Rg, SASA, and MM-PBSA. In addition, MD simulation was performed in YASARA Structure software with a 100 ns trajectory, and the all-atom force fields AMBER14 and SASA for the complexes.

### Solvent-accessible surface area analysis

**Figure 7:**
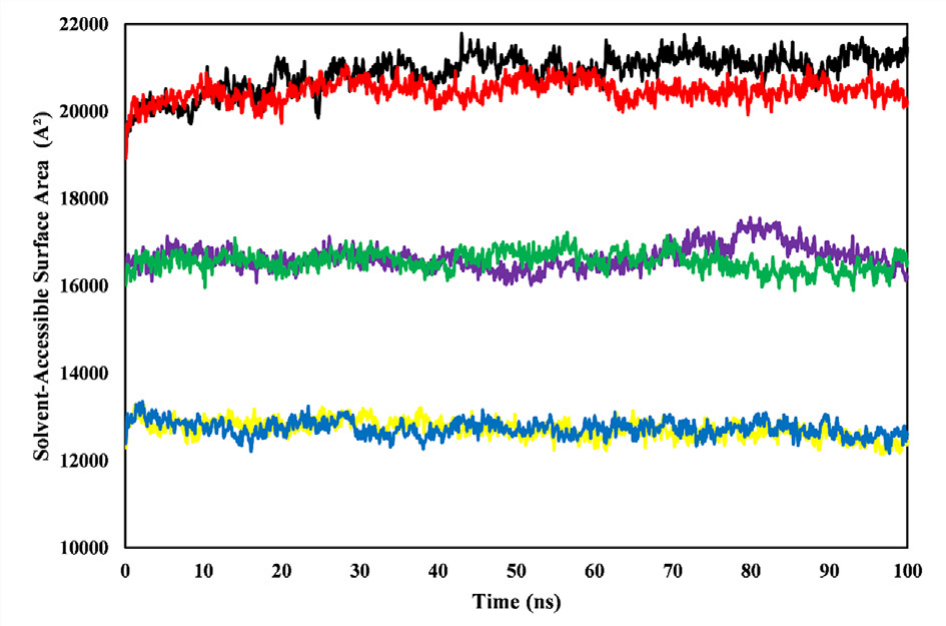
SASA as a function of simulation time, depicted for the ligand–enzyme complexes after 100 ns MD simulations. The plot includes complexes of catechin-AChE (black), galantamine-AChE (red), chlorogenic acid-TACE (purple), aryl-sulfonamide-TACE (green), chlorogenic acid-BACE1 (yellow), and 2-imino-3-methyl-5,5-diphenylimidazolidin-4-one-BACE1 (blue).

SASA analysis indicated the stability of the control drugs and the chlorogenic acid-BACE1 complex in 100 ns MD simulations. The complexes showed minimal fluctuations in SASA values, thus suggesting minimal changes in the solvent exposure of the protein surface after ligand binding (Figure 7). Chlorogenic acid-TACE showed a transient increase in SASA at approximately 80 ns, thus indicating a brief alteration in solvent exposure, but stability recovered by the end of the simulation. Meanwhile, catechin-AChE showed similar stability, with transient deviations at 24 ns and 44 ns. The average SASA values for catechin-AChE and galantamine-AChE were 21,494.72 Å^2^ and 20,076.72 Å^2^, respectively. Chlorogenic acid-TACE and aryl-sulfonamide-TACE had average SASA values of 16,097.03 Å^2^ and 16,759.12 Å^2^, respectively. For BACE1 complexes, chlorogenic acid and 2-imino-3-methyl-5,5-diphenylimidazolidin-4-one showed average SASA values of 12,635.35 Å^2^ and 12,563.41 Å^2^, respectively.

### Root mean square deviation analysis

**Figure 8:**
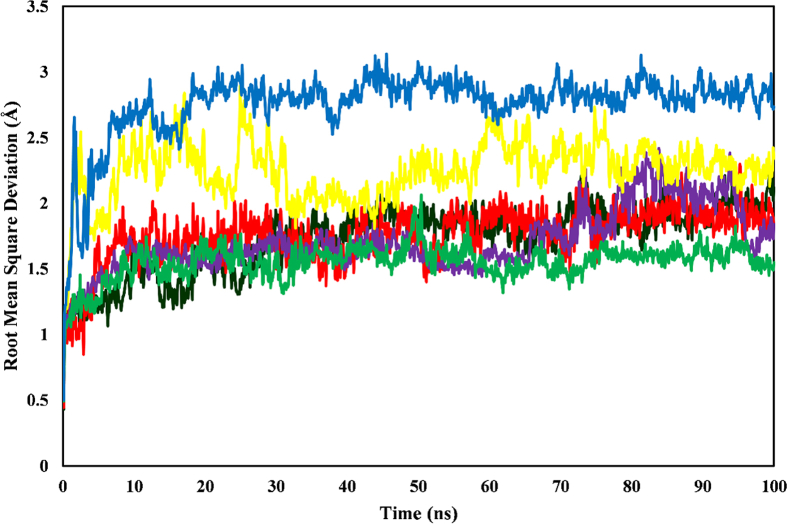
RMSD trajectories of the ligand–enzyme complexes, monitored over a 100 ns MD simulation. The complexes include catechin-AChE (black), galantamine-AChE (red), chlorogenic acid-TACE (purple), aryl-sulfonamide-TACE (green), chlorogenic acid-BACE1 (yellow), and 2-imino-3-methyl-5,5-diphenylimidazolidin-4-one-BACE1 (blue).

Analysis of RMSD trajectories indicated that all ligand–enzyme complexes except chlorogenic acid-BACE1 and aryl-sulfonamide-TACE showed stability in the 100 ns MD simulation (Figure 8). These complexes' minimal RMSD fluctuations suggested relatively minimal structural deviations throughout the procedure. The chlorogenic acid-BACE1 complex showed greater RMSD fluctuations than the other complexes, but the average value remained relatively low, at 2.241 Å. Despite maintaining stability, the chlorogenic acid-BACE1 complex underwent transient conformational adjustments during the simulation. In line with these observations, the 2-imino-3-methyl-5,5-diphenylimidazolidin-4-one-BACE1 complex showed the highest average RMSD value (2.776 Å) among all complexes investigated. In contrast, the control drugs galantamine-AChE (1.752 Å) and aryl-sulfonamide-TACE (1.550 Å) had significantly lower average RMSD values indicating high structural stability in the simulation, and the average for catechin-AChE was 2.107 Å.

### Radius of gyration analysis

**Figure 9:**
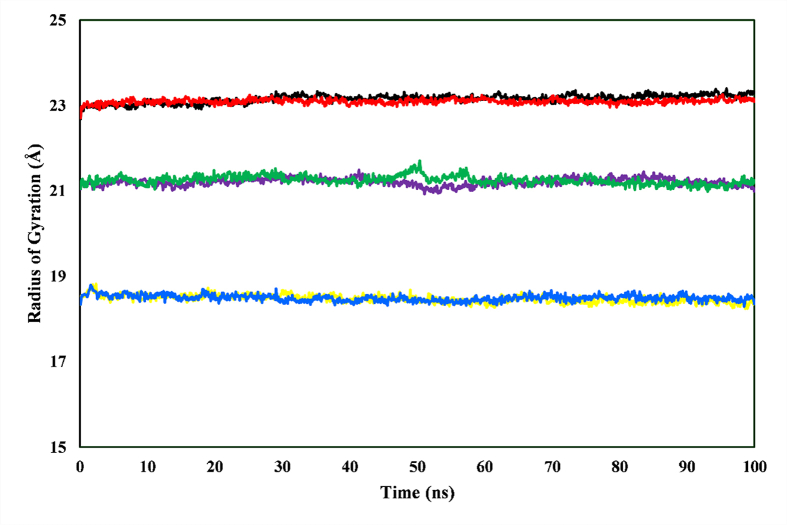
Rg trajectories of the ligand–enzyme complexes, monitored throughout a 100 ns MD simulation. The complexes include catechin-AChE (black), galantamine-AChE (red), chlorogenic acid-TACE (purple), aryl-sulfonamide-TACE (green), chlorogenic acid-BACE1 (yellow), and 2-imino-3-methyl-5,5-diphenylimidazolidin-4-one-BACE1 (blue).

Rg trajectories were analyzed to assess the compactness of the ligand–enzyme complexes in 100 ns MD simulations (Figure 9). In addition, Rg values reflected the compactness of the protein structure, with lower values indicating a more compact conformation. The complexes maintained relatively stable Rg values in the simulation, thus suggesting minimal unfolding or significant conformational changes. However, aryl-sulfonamide-TACE showed a slightly higher average value (21.217 Å) than chlorogenic acid-TACE (21.092 Å), thereby suggesting a marginally less compact structure. The Rg values of catechin-AChE (23.23 Å) and chlorogenic acid-BACE1 (18.405 Å) were slightly higher than those of the respective control drugs, galantamine-AChE (23.051 Å) and 2-imino-3-methyl-5,5-diphenylimidazolidin-4-one-BACE1 (18.368 Å). Subtle differences were observed in the compactness of the ligand-bound conformations compared with the control drugs.

### Molecular mechanics Poisson–Boltzmann surface area calculation method

**Figure 10:**
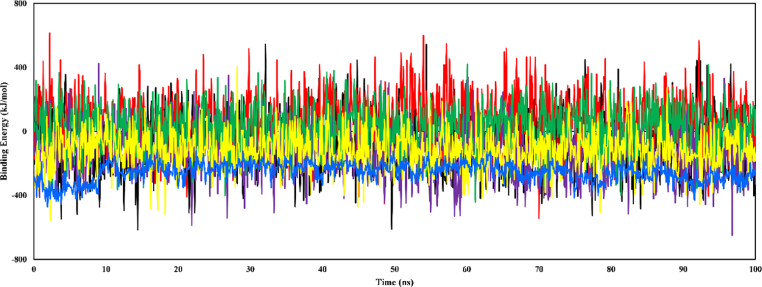
Estimated MM-PBSA binding free energies of the ligand–enzyme complexes, presented for the 100 ns MD simulation. The complexes include galantamine-AChE (red), chlorogenic acid-TACE (purple), aryl-sulfonamide-TACE (green), chlorogenic acid-BACE1 (yellow), and 2-imino-3-methyl-5,5-diphenylimidazolidin-4-one-BACE1 (blue).

MM-PBSA binding free energy analysis was used to evaluate the binding interactions of the ligand–enzyme complexes in 100 ns MD simulations (Figure 10). The complexes showed favorable ligand–enzyme interactions, as evidenced by negative average binding free energies ranging from −10.482 kJ/mol (galantamine-AChE) to −337.172 kJ/mol (chlorogenic acid-TACE). In addition, the calculated average binding free energies for catechin-AChE, galantamine-AChE (control drug), chlorogenic acid-TACE, aryl-sulfonamide-TACE (control drug), chlorogenic acid-BACE1, and 2-imino-3-methyl-5,5-diphenylimidazolidin-4-one-BACE1 (control drug) were −310.126 kJ/mol, −10.482 kJ/mol, −337.172 kJ/mol, −15.176 kJ/mol, −145.798 kJ/mol, and −276.758 kJ/mol, respectively. Positive fluctuations in the binding free energy trajectories in the simulations for most complexes suggested dynamic interactions between the ligands and enzymes. The galantamine-AChE, aryl-sulfonamide-TACE, and chlorogenic acid-BACE1 complexes showed stronger preferences for the lower energy region below 0 kJ/mol than the others in the respective groups. Therefore, these ligand–enzyme interactions might be relatively stable.

### Root mean square fluctuation analysis

**Figure 11:**
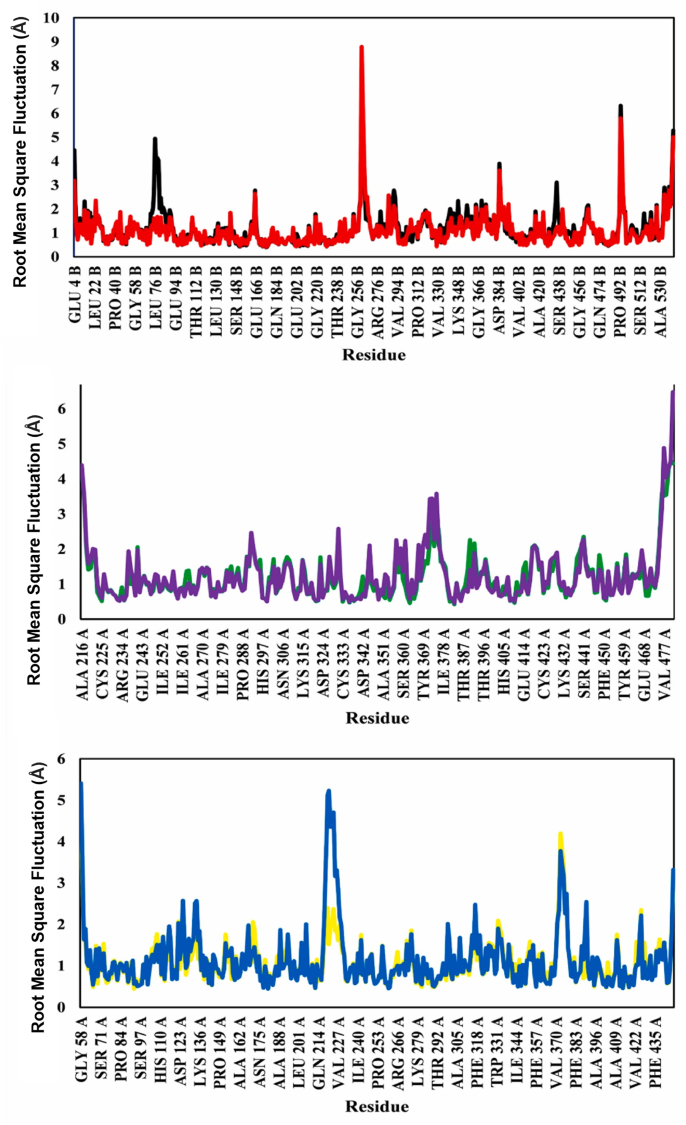
RMSF plot of catechin-AChE (black), galantamine-AChE (red), chlorogenic acid-TACE (purple), aryl-sulfonamide-TACE (green), chlorogenic acid-BACE1 (yellow), and 2-imino-3-methyl-5,5-diphenylimidazolidin-4-one-BACE1 (blue).

RMSF profiles showed similar stability in the 100 ns MD simulations for all complexes, in line with the results from RMSF analysis (Figure 11). RMSF characterized the average fluctuation of each amino acid residue in a protein structure. Low values indicated minimal movement of the residues, thus suggesting a stable protein structure. The observed RMSF values for all complexes were below 1.4 Å, thereby indicating the stability of the ligand–enzyme systems during the simulation.

## Discussion

AD is a neurodegenerative disorder characterized by aggregation of amyloid plaques and protein tangles in the brain. In this context, BACE1 inhibition may provide a strategy to decrease the production of amyloid-β peptides, a major component of the amyloid plaques that accumulate in the brains of affected individuals. Docking simulation revealed that chlorogenic acid formed favorable interactions in the BACE1 binding pocket. Therefore, by inhibiting BACE1 activity, these compounds might potentially slow the formation and progression of amyloid plaques, a hallmark of AD.^32^^,^^33^ Inhibition of AChE was predicted to increase brain levels of acetylcholine, a neurotransmitter crucial for memory and cognitive function, thus potentially improving cognitive function in affected patients. In addition, TACE processed various proteins, including those contributing to the neuroinflammatory response observed in AD. Although the specific role of TACE in AD pathogenesis remains under investigation, inhibition by compounds such as chlorogenic acid has been shown to offer benefits by decreasing neuroinflammation.^34^^,^^35^ Our study used a novel *in silico* method to explore potential therapeutic candidates for AD derived from natural compounds isolated from *S. columbaria*. Computational methods were leveraged for drug discovery while focusing on the previously unexplored source *S. columbaria*.

The identified phytochemicals were evaluated according to Lipinski's rule of five to assess drug-likeness properties. This widely used rule predicts compounds' absorption, distribution, metabolism, excretion, and toxicity (ADMET) profiles according to their physicochemical properties. Specifically, Lipinski's rule suggests that compounds with a molecular weight less than 500 Da, a logP value below 5, no more than ten hydrogen bond acceptors, no more than five hydrogen bond donors, and fewer than ten rotatable bonds show favorable drug-like characteristics. These parameters influence compounds' ability to be absorbed, to be distributed in the body, to be metabolized and excreted, and to potentially cause toxicity (collectively known as the ADMET profile). According to Lipinski's rule, favorable properties suggest a high likelihood of a compound's successful navigation of these processes in the body. The phytochemicals inconsistent with the rule violated only logP >3, thus potentially indicating poor solubility and bioavailability, and negatively affecting their efficacy and safety as drugs.^26^ *In silico* acute oral toxicity prediction was performed for all ligands to evaluate potential safety concerns. This method categorized compounds according to predicted toxicity 24 h after oral administration. Classes 1 and 2 suggest potential toxicity, whereas classes 3 and 4 suggest a non-toxic profile.^27^ *In silico* toxicity prediction offers substantial advantages over traditional animal testing by promoting efficiency and decreasing the use of experimental animals.^36^ During *in silico* analysis, all investigated compounds, specifically catechin and chlorogenic acid, were predicted to be non-toxic, and consequently were evaluated through docking simulations. The compounds had no identified drawbacks that might affect their development as drugs.

After *in silico* ADMET and toxicity evaluation, all qualifying chemical compounds from *S. columbaria* were subjected to molecular docking simulation. Molecular docking is a cornerstone bioinformatics method used to predict binding modes and estimate small molecules' affinities (energies) with target proteins. The simulations provided valuable insights into the potential interactions between *S. columbaria* phytochemicals and the three enzymes of interest in AD: AChE, BACE1, and TACE. Docking simulation is a widely used tool in computer-aided drug discovery for identifying novel lead compounds with promising therapeutic potential.^37^ In addition, the docking protocol was validated by calculation of the RMSD between the docked ligand pose and the reference structure from the co-crystallized complex. A docking pose was considered successful when the RMSD was below the predefined threshold of 2.0 Å, thus indicating high accuracy.^38^ All receptors (AChE, BACE1, and TACE) had RMSD values below the 2.0 Å cutoff (Figure 5; 0.2709 Å for AChE, 1.555 Å for BACE1, and 1.486 Å for TACE). After molecular docking simulation, a screening method was used to identify the top-scoring molecules according to their predicted binding affinities toward each of the three target enzymes. In molecular docking, the binding affinity is a key parameter used to estimate the affinity energy between a ligand and a protein. A more strongly negative binding affinity value indicates a more favorable and stable ligand-protein complex. *In silico* methods, as reported by Irsal,^28^ are valuable in prioritizing promising drug candidates.

The docking results for AChE and BACE1 showed favorable binding affinities for catechin and chlorogenic acid. However, the binding affinity values were not significantly lower than those of the established drugs galantamine-AChE and aryl-sulfonamide-BACE1 (Figure 6). Specifically, the binding affinity values for catechin-AChE and chlorogenic acid-AChE complexes were −9.742 kcal/mol and −9.063 kcal/mol, as compared with −10.526 kcal/mol for galantamine-AChE. BACE1, catechin, and chlorogenic acid showed binding affinity values of −8.000 kcal/mol and −8.646 kcal/mol, whereas the control drug aryl-sulfonamide presented a value of −8.983 kcal/mol. These results suggested that structural optimization of *S. columbaria*-derived compounds might be necessary to achieve superior inhibitory potential to that of existing therapies for AChE and BACE1. However, the broader health benefits associated with catechin, such as its antioxidant, anti-inflammatory, and anti-microbial properties, must be further investigated.^39^ These additional attributes might contribute to the therapeutic potential of catechin, despite its lower binding affinity value for AChE inhibition.

Our results indicated the potential of *S. columbaria* as a source of novel drug candidates. Chlorogenic acid showed a remarkable binding affinity toward TACE (−9.381 kcal/mol), exceeding that of the control drug aryl-sulfonamide (−8.791 kcal/mol). Catechin also showed promising binding to TACE, with a binding affinity value of −8.783 kcal/mol. Chlorogenic acid has been reported to have neuroprotective effects and to promote brain function.^40^ Although some compounds did not outperform the controls in terms of binding affinity toward AChE and BACE1, the identified ligands for each enzyme may be promising candidates for further development.

The number of hydrogen bonds and hydrophobic interactions significantly affects the strength and stability of ligand-receptor binding. Non-covalent and hydrophobic interactions significantly influence the strength and stability of ligand-receptor binding. In addition, 2D visualization identifies two types of hydrogen bonds, conventional and carbon-hydrogen bonds, whereas hydrophobic interactions include pi and alkyl.^41^ Conventional hydrogen bonds from hydrogens with partial positive charges interacting with electronegative atoms, such as oxygen, nitrogen, or fluorine, are stronger than carbon-hydrogen bonds between aromatic CH groups and amino acid residue oxygens.^42^ These interactions collectively have a crucial role in determining binding affinity and specificity. Our study supported predictions that hydrogen bonds enhance ligand–receptor interactions.^43^ The control drugs, galantamine, aryl-sulfonamide, and 2-imino-3-methyl-5,5-diphenylimidazolidin-4-one, formed hydrogen bonds at the active sites. Therefore, hydrogen bonding appeared to contribute to the efficacy of the control drugs. Hydrophobic interactions appeared to indicate the degree of stability of ligand–receptor complexes. Moreover, these interactions aid in activating biomolecular responses triggered by protein folding, in which non-polar residues, such as leucine, valine, isoleucine, and alanine, are buried in the protein's interior and shielded, thereby minimizing water interactions.^42^

MD simulation of the binding pockets of AChE, BACE1, and TACE was conducted to provide insights for drug discovery. In addition, AMBER14 was used as the computational tool for simulation. The force field included a comprehensive set of parameters delineating atom interactions in a molecule, comprising bond stretching, angle bending, and non-bonded interactions. AMBER14 offers a suite of functionalities, such as input file preparation, simulation execution, analysis of results, and visualization of molecular structures and interactions. This software facilitates the exploration of biomolecule dynamics and properties under diverse conditions and environments in biochemistry and drug discovery.^31^^,^^44^ RMSD, SASA, and Rg were acquired after the execution of md_analyze. RMSF was obtained through md_analyze, whereas molecular mechanics was calculated with MM-PBSA with md_analyzebindingenergy.

The SASA graph indicated the compactness of the target protein after interaction with an inhibitor, primarily through hydrophobic interactions.^41^ Catechin-AChE (23.23 A^2^), aryl-sulfonamide-TACE (21.217 A^2^), and chlorogenic acid-BACE1 (18.405 A^2^) showed the highest SASA, as compared with the counterparts in each respective enzyme (Figure 7). This observation indicated a potential decrease in the active site volume and hydrophobicity with increased SASA, thus potentially restricting the flexibility of the ligand in the active site crucial for optimal interactions. Increased hydrophilicity of the active site might lead to inferior ligand binding, thereby affecting ligand binding characteristics and selectivity. The ligand complexes maintained stable SASA values, thereby indicating minimal fluctuations in surface area and substantial rigidity.

During the 100 ns and 5 ns simulations, all complexes showed stable RMSD movement (Figure 8). The complexes other than those with 2-imino-3-methyl-5,5-diphenylimidazolidin-4-one-BACE1 maintained an interaction stability below 2.5 Å.^4^ The lower values observed for the ligand-docked proteins than the unbound proteins further supported stable and well-defined ligand-protein interactions.

Rg is a fundamental metric for characterizing the sizes of chain molecules and a key indicator of protein compactness and flexibility in biological contexts. In this study, this variable enabled the comparison of the protein structures relative to the hydrodynamic radius, an experimentally observable parameter. According to Ghahremanian et al., insights derived from measurements can substantially contribute to understanding of the dynamic interplay between proteins and their surrounding environment.^45^ The complexes bound to AChE and BACE1 maintained stability during the simulation period. However, the reference compound for TACE, aryl-sulfonamide, showed slight fluctuations between 48 and 58 ns. The phytocompound chlorogenic acid, bound to TACE, remained stable in the simulation (Figure 9). Catechin-AChE, aryl-sulfonamide-TACE, and chlorogenic acid-BACE1 showed higher Rg values than the counterparts in each respective enzyme. Elevated Rg values indicate looser protein packing, thus suggesting higher protein conformation flexibility.

YASARA's built-in macros were used to conduct calculations, where positive energy indicated stronger binding. In addition, stronger binding and compact interactions were indicated by higher binding energy values in MM-PBSA calculations.^46^ The minimal RMSF values suggested that the compound complexes maintained stable conformations in the simulations, thereby indicating favorable interactions with the investigated enzymes (Figure 11).^30^

MD simulation provides insights into the stability of ligand–protein complexes and consequently the pharmacodynamics of the compounds. A highly stable complex, indicated by low RMSD fluctuations and minimal structural changes (Rg), suggests tight binding to the enzyme's active site. Although potent enzyme inhibition might result, other effects might include prolonged drug action or difficulty in drug clearance by the body, thus potentially causing adverse effects. In contrast, high RMSD fluctuations or Rg values in the simulation might signify readily dissociated ligands, thus potentially translating to a short duration of action for the drug and a need for frequent dosing to maintain therapeutic effects.^47^ In this study, chlorogenic acid showed a relatively stable complex with minimal fluctuations throughout the simulation. The properties, coupled with the high binding energy determined from MM-PBSA calculations, indicated a potentially potent inhibitory effect on TACE and BACE1 activity. However, further investigation is needed to determine whether this strong binding might translate to extended drug action or potential adverse effects.

Chlorogenic acid is a widespread phenolic compound in plants including coffee, fruits, vegetables, and traditional Chinese herbal medicines. This study explored the potential of chlorogenic acid to serve as an anti-AD agent, according to its ability to interact with three key enzymes associated with AD: AChE, BACE1, and TACE. Beyond its potential role in AD treatment, chlorogenic acid exhibits a broad spectrum of biological activities, including antioxidant, hepatoprotective (liver protection), nephroprotective (kidney protection), antibacterial, antitumor, metabolic regulatory (sugar and lipid metabolism), anti-inflammatory, and neuroprotective effects.^7^ In the context of TCM, this compound is often applied in TCM injections, and it is the main active component in various herbal formulations used for antibacterial and anti-inflammatory purposes. Historically, the effectiveness of TCM has been attributed to its inherent antioxidant, anti-inflammatory, and antitumor properties.^11^

This study described a screening method to explore potential treatments for patients with AD. According to Poon et al.^48^ the use of animal models must be considered, because AD is a neurodegenerative disorder to which many molecular pathways are susceptible, including neuroinflammation, the immune response, neuroplasticity, and neurotrophic factors. By studying the weaknesses or points of susceptibility in biological systems, more effective drugs may be developed.

This *in silico* investigation identified *S. columbaria*-derived chlorogenic acid compounds with binding affinity toward TACE and BACE1. Catechin also has potential in the development of anti-AD drugs. Moreover, the top compounds did not violate Lipinski's rule of five, and had toxicity classes of 4 (catechin) and 3 (chlorogenic acid). Our findings may substantially contribute to the development of novel AD treatments, after validation through *in vitro* and *in vivo* studies.

## Limitations

This study has several limitations. We conducted a comprehensive investigation of the potential of compounds from *S. columbaria* as anti-AD agents by using computational methods. However, our reliance on an *in silico* method introduced uncertainties regarding the accuracy and reliability of the results, thus necessitating future validation through experimental studies. Moreover, selecting and characterizing ligands extracted from *S. columbaria* may overlook other beneficial compounds, potentially limiting the scope. Simplified protein models in molecular docking and MD simulations might not fully capture the dynamic nature of protein–ligand interactions, thus leading to potential predictive inaccuracies. The sensitivity of simulation to various parameters indicates the importance of careful optimization to ensure reliable outcomes. Finally, the lack of experimental validation prevented the confirmation of predicted interactions and inhibitory activity of the identified compounds. This study offers valuable insights by integrating multiple computational methods, and analyzing binding interactions and molecular properties. The identified compounds showed promise as potential anti-AD agents, thereby laying a foundation for further experimental validation and drug development efforts.

## Conclusions

This study provided valuable insights into the potential of *S. columbaria* compounds as inhibitors of three enzymes implicated in AD: AChE, BACE1, and TACE. Several promising natural compounds from *S. columbaria* were identified to have favorable binding affinities toward the enzymes. Catechin exhibited a binding affinity toward AChE (−9.742 kcal/mol) similar to that of the control drug. Similarly, chlorogenic acid strongly was found to bind BACE1 (−9.381 kcal/mol) and to have the second-best affinity to TACE (−8.646 kcal/mol), near that of the control drug. Catechin surpassed the control drug galantamine in terms of binding affinity and targeted different interaction sites on AChE. Chlorogenic acid also demonstrated stronger binding affinity than the control drug toward TACE, potentially through a distinct binding mechanism. In addition, MD simulation provided stability assessments for the protein-ligand complexes. Whereas most complexes exhibited overall stability, aryl-sulfonamide-TACE showed deviations requiring further investigation. These results warrant further exploration of *S. columbaria* as a source of natural anti-AD agents. The identified compounds, particularly catechin and chlorogenic acid, may be promising candidates for future *in vitro* and *in vivo* studies to validate their potential as therapeutic interventions for AD.

## Source of funding

This research did not receive any specific grant from funding agencies in the public, commercial, or not-for-profit sectors.

## Conflict of interest

The authors have no conflict of interest to declare.

## Ethical approval

Not applicable; there are no ethical issues.

## Authors contributions

RAPI: Conceptualization; data curation; validation; investigation; methodology; resources; formal analysis; writing—original draft; writing—review & editing. GMG: Conceptualization; data curation; validation; investigation; methodology; resources; formal analysis; writing—original draft; writing—review & editing. MAD: Data curation; resources; validation; investigation; writing—review & editing. TFM: Review & editing. FC: Supervision; review & editing. All authors have critically reviewed and approved the final draft and are responsible for the content and similarity index of the manuscript.
